# Plasma metabolites as possible biomarkers for diagnosis of breast cancer

**DOI:** 10.1371/journal.pone.0225129

**Published:** 2019-12-03

**Authors:** Jiwon Park, Yumi Shin, Tae Hyun Kim, Dong-Hyun Kim, Anbok Lee

**Affiliations:** 1 Department of Surgery, Busan Paik Hospital, College of medicine, Inje University, Busan, Korea; 2 Department of Pharmacology, College of medicine, Inje University, Busan, Korea; Indian Institute of Chemical Technology, INDIA

## Abstract

Metabolomic approaches have been used to identify new diagnostic biomarkers for various types of cancers, including breast cancer. In this study, we aimed to identify potential biomarkers of breast cancer using plasma metabolic profiling. Furthermore, we analyzed whether these biomarkers had relationships with clinicopathological characteristics of breast cancer. Our study used two liquid chromatography-mass spectrometry sets: a discovery set (40 breast cancer patients and 30 healthy controls) and a validation set (30 breast cancer patients and 16 healthy controls). All breast cancer patients were randomly selected from among stage I–III patients who underwent surgery between 2011 and 2016. First, metabolites distinguishing cancer patients from healthy controls were identified in the discovery set. Then, consistent and reproducible metabolites were evaluated in terms of their utility as possible biomarkers of breast cancer. Receiver operating characteristic (ROC) analysis was applied to the discovery set, and ROC cut-off values for the identified metabolites derived therein were applied to the validation set to determine their diagnostic performance. Ultimately, four candidate biomarkers (L-octanoylcarnitine, 5-oxoproline, hypoxanthine, and docosahexaenoic acid) were identified. L-octanoylcarnitine showed the best diagnostic performance, with a 100.0% positive predictive value. Also, L-octanoylcarnitine levels differed according to tumor size and hormone receptor expression. The plasma metabolites identified in this study show potential as biomarkers allowing early diagnosis of breast cancer. However, the diagnostic performance of the metabolites needs to be confirmed in further studies with larger sample sizes.

## Introduction

Breast cancer is the most common cancer in females worldwide. In developed countries, one out of eight women will develop invasive breast cancer during their lifetime [1]. Advanced breast cancer has a poor prognosis, but early diagnosis and appropriate treatment can improve the disease course; the long-term survival of patients with breast cancer depends on the disease stage at the time of diagnosis [2]. Many studies have been conducted on the early detection of breast cancer, which can dramatically increase the survival rate [3]. Several screening tools are available, including mammography and ultrasonography. Mammography is widely used for early screening of breast cancer; however, it relatively insensitive, especially for dense breast tissue, while ultrasonography is highly dependent on the ability of the practitioner and is not suitable for primary screening test due to its high cost. Even using these imaging techniques, approximately 2% of patients who undergo breast cancer screening have false-negative results [4]. Plasma tumor markers are being researched extensively to aid diagnosis of breast cancer; there is a need for a test with high sensitivity and specificity for early diagnosis.

Metabolomic approaches have been used to identify possible markers and key metabolic pathways in various types of cancers. Proliferating cancer cells show different metabolic behavior compared to normal differentiated cells [5]. Metabolomic analyses have been actively conducted on urine, plasma, and tissue samples against this background. There are two widely used techniques for identification of metabolites: mass spectrometry (MS) and nuclear magnetic resonance (NMR) spectroscopy [6]. Both techniques are used extensively in metabolomic studies, and they each possess unique advantages and limitations. NMR spectroscopy allows for quantitative analysis and does not require extra steps for sample preparation, such as separation or derivatization [7]. Although the sensitivity of NMR spectroscopy has increased significantly, MS remains more sensitive [8]. However, unlike NMR spectroscopy, MS requires initial separation of metabolites using chromatography, and is therefore usually coupled with gas chromatography (GC) or liquid chromatography (LC) [9]. MS is known to have both high sensitivity and selectivity, and allows for simultaneous analysis of hundreds of metabolites in a biological specimen [10]. LC-MS-based metabolomics is the optimal approach for discovering biomarkers and exploring metabolites [11].

We conducted LC-MS experiments using two data sets: a discovery set and a validation set. In the discovery set, we aimed to identify metabolites that distinguished cancer patients from healthy controls. In the discovery set, consistent and reproducible metabolites that could ultimately be used for screening breast cancer were evaluated. Furthermore, receiver operating characteristic (ROC) analysis was performed on the discovery set metabolites, and ROC cut-off values derived therein for the identified metabolites were applied to the validation set to determine their diagnostic performance.

## Material and methods

### Selection of patients and collection of plasma samples

The study protocol was approved by the Institutional Review Board of Inje University Busan Paik Hospital, Busan, Korea. Plasma samples were provided by the Inje Biobank of Inje University Busan Paik Hospital and the Biobank of Chungnam National University Hospital.

### Discovery set

Forty breast cancer patients and thirty healthy controls were included in the discovery set. Breast cancer patients were diagnosed and surgically treated at Inje University Busan Paik Hospital from 2011 to 2016. Fifteen stage I–II patients were randomly selected, as well as ten stage III patients. Patients who received neoadjuvant chemotherapy were excluded. Healthy controls were recruited from among those without a history of other diseases, including malignancies.

### Validation set

Thirty breast cancer patients, including ten stage I–III patients, and sixteen healthy controls (selected in the same manner as per the validation set) were included in the validation set.

Blood samples were collected from the breast cancer patients on the day before surgery. Fasting blood samples were collected in the morning from healthy controls who signed informed consent forms. All blood samples were stored in K2-EDTA vacutainer tubes and immediately cooled in a refrigerator (4°C). Within 2 hours of collection, they were centrifuged at 3,000 × *g* for 10 min at 4°C. Then, the samples were transferred into new vials and immediately stored frozen (-80°C) until preparation. Pathological analysis of surgically resected specimens was performed.

### Sample preparation

Three volumes of acetonitrile containing 5 μg/mL cholic acid-d5 (internal standard) were added to each 50 μL plasma sample. The mixture was then vortexed and centrifuged at 13,200 rpm for 5 min at 4°C. The supernatant was used for high-performance liquid chromatography (HPLC) analysis. Cholic acid-d5 was purchased from Toronto Research Chemicals (Toronto, Canada).

### Chromatographic separation and mass analysis

Analyses were conducted using an Agilent 6530 quadrupole time-of-flight mass spectrometer (Agilent Technologies, Santa Clara, CA, USA) coupled with an Agilent 1200 series HPLC system. The separations were performed with a BEH C_18_ column (100 × 2.1 mm, 1.7 μm; Waters, Milford, MA, USA) and a ZIC-HILIC column (100 × 2.1 mm, 3.5 μm; Merck, Darmstadt, Germany). For the BEH C_18_ column, the mobile phase was 0.1% formic acid in water (A) and 0.1% formic acid in acetonitrile (B) at a flow rate of 0.4 mL/min. The gradient conditions were as follows: 2% mobile phase B maintained for 1 min initially, and then increased to 20% at 3 min and 90% at 8 min, and finally maintained for 6 min. The temperature of the column and autosampler was 35°C and 4°C, respectively, and the injection volume was 3 μL. For the ZIC-HILIC column, the mobile phase was 10 mM ammonium acetate in 5/95 acetonitrile/water (A) and 10 mM ammonium acetate in 95/5 in acetonitrile/water (B) at a flow rate of 0.5 mL/min. The gradient conditions were as follows: 1% mobile phase A maintained for 1 min initially, and then increased to 50% at 15 min, and finally maintained for 2 min. The temperature of the column and autosampler was 40°C and 4°C, respectively, and the injection volume was 5 μL. Electrospray ionization was performed in both positive ion and negative ion modes for the BEH column, but only in negative ion mode for the ZIC-HILIC column. The sheath gas flow rate was set to 11 L/min. The drying gas flow rate was set to 12 L/min at 350°C. The nebulizer temperature was maintained at 350°C. The capillary voltage was set to 4,000 V in positive mode and -4,000 V in negative mode, and the fragmentor was set to 110 V. All data were acquired in a scan range of m/z = 50 to 1,000 in centroid mode. Reference compounds (C_18_H_18_O_6_N_3_P_3_F_24_; [M + H]^+^ = 922.0098 and [M + formate]^-^ = 966.0007) were used to adjust the mass during analysis. Auto tandem mass spectrometry (MS/MS) analysis was conducted for peak identification with a collision energy of 30 eV.

### Data processing and analysis

Mass data acquisition and processing were conducted as described previously [12]. Integrated mass spectrometric data were initially converted into mzXML format and processed using the XCMS package in R software (ver. 3.1.0; R Development Core Team, Vienna, Austria) for peak detection, alignment, and integration. The data were then subjected to multivariate analysis using the SIMCA-P11.5 software package (Umetri AB, Umea, Sweden). For data normalization, LOESS fitting was applied in the R environment using NOREVA (http://idrb.zju.edu.cn/noreva/). All normalized peaks were subjected to multivariate analysis. Principle component analysis (PCA) was performed to visualize patterns and groupings, and partial least squares-discriminant analysis (PLS-DA) was conducted to determine metabolites distinguishing the cancer patients from the control subjects, based on their variable importance in projection (VIP) values, using the SIMCA-P11.5 software package (Umetris AB). Highly ranked metabolites according to PCA and the PLS-DA VIP values were subjected to further structural analysis. An in-house database, as well as the Human Metabolome Database (HMDB; http://www.hmdb.ca), the Metlin database (metlin.scripps.edu), and the Kyoto Encyclopedia of Genes and Genomes database (KEGG; http://www.genome.jp/kegg/ or http://www.kegg.jp/) were used to identify metabolites with potential as biomarkers of breast cancer.

### Statistical analysis

All statistical analyses were performed using SPSS statistical software for Windows (version 23.0; IBM Corp., Armonk, NY, USA). The nonparametric Kruskal-Wallis and Mann-Whitney U tests were used to analyze differences between the cancer and control groups. A *p*-value of < 0.05 was considered statistically significant. To evaluate the sensitivity and specificity of the potential biomarkers, ROC curves were plotted using MedCalc software (version 16.4.3; MedCalc Software, Ostend, Belgium).

## Results

### Characteristics of patients and healthy controls

A total of 116 females (70 breast cancer patients and 46 healthy controls) were included in this study. The 46 healthy controls were all women, with a mean age of 54.6 years (range: 45–59 years). The mean age of the breast cancer group was 59 years (range: 34–92 years). Among the 70 breast cancer patients, there were 25 stage I cases (35.7%), 25 stage II cases (35.7%), and 20 stage III cases (28.6%).

The subjects were divided into a discovery set of 70 subjects and a validation set of 46 subjects based on the cancer stage distribution. Table 1 shows the clinicopathological characteristics of the breast cancer patients and healthy controls in the discovery and validation sets.

**Table 1:** Clinicopathological characteristics of breast cancer patients and healthy controls in the discovery and validation sets.

	Discovery set		Validation set	
	Cancer (n = 40)	Healthy (n = 30)	Cancer (n = 40)	Healthy (n = 16)
**Age (years), median**	55.5	53.0	57	56.5
**Histological grade**				
Low	6 (15.0%)		4 (13.3%)	
Intermediate	12 (30.0%)		9 (30.0%)	
High	22 (55.0%)		17 (56.7%)	
**Tumor size**				
≤ 2 cm	21 (52.5%)		13 (43.3%)	
> 2 cm	19 (47.5%)		17 (56.7%)	
**LN metastasis**				
Negative	23 (57.5%)		16 (53.3%)	
Positive	17 (42.5%)		14 (46.7%)	
**ER status**				
Negative	15 (37.5%)		10 (33.3%)	
Positive	25 (62.5%)		20 (66.7%)	
**PR status**				
Negative	18 (45.0%)		13 (43.3%)	
Positive	22 (55.0%)		17 (56.7%)	
**HER2 status**				
Negative	27 (67.5%)		22 (73.3%)	
Positive	13 (32.5%)		8 (26.7%)	
**Ki-67**				
Low	6 (15.0%)		4 (13.3%)	
Intermediate	11 (27.5%)		10 (33.3%)	
High	23 (57.5%)		16 (53.3%)	
**TNM stage**				
I	15 (37.5%)		10 (33.3%)	
II	15 (37.5%)		10 (33.3%)	
III	10 (25.0%)		10 (33.3%)	

LN; lymph node, ER; estrogen receptor, PR; progesterone receptor; TNM, tumor node metastasis.

Plasma metabolite profiles of the breast cancer patients

**Fig 1:**
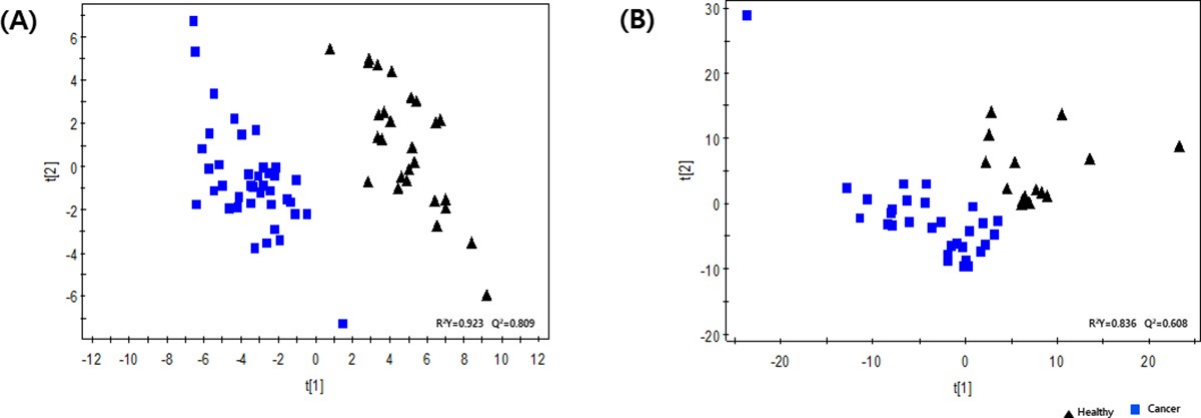
Partial least squares-discriminant analysis score plots of healthy subjects (▲) and cancer patients (■) in the discovery (A) and validation sets (B). Clear separation between the cancer and healthy groups was observed, with all healthy controls being distributed on the right side and most of the breast cancer patients being distributed on the left side in the discovery set. Clear separation was also observed between the two groups in the validation set.

PLS-DA was conducted on the discovery set. The PLS-DA model had one predictive component and two orthogonal components (R_2_X = 0.077, R_2_Y = 0.923, and Q_2_Y = 0.809). Clear separation between the cancer and control groups was observed, with all healthy controls being distributed on the right side and most of the breast cancer patients being distributed on the left side (Fig 1A). In the same manner, PLS-DA was performed on the validation set. Clear separation between the groups was again observed, and there was one predictive component and two orthogonal components (R2X = 0.163, R2Y = 0.836, and Q2Y = 0.608) (Fig 1B).

### Selection of candidate metabolic biomarkers

**Fig 2:**
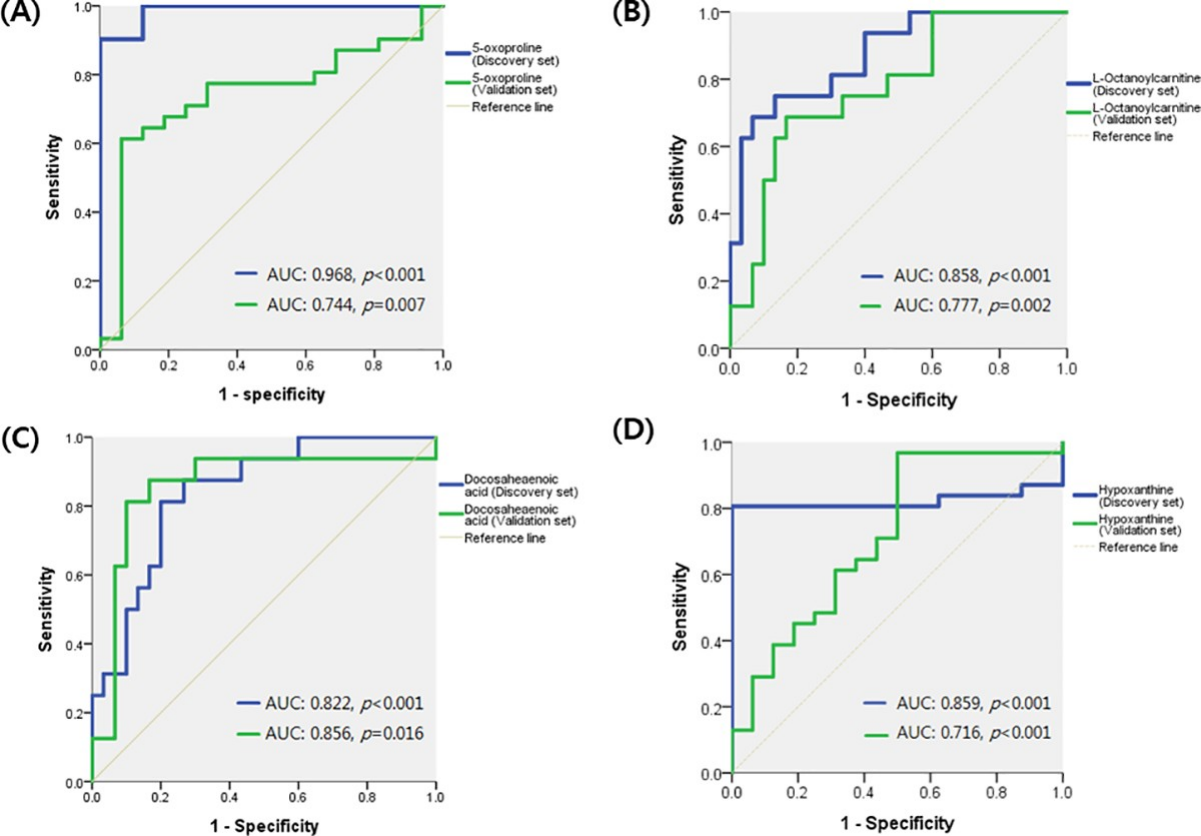
Receiver operating characteristic curves of candidate biomarkers in the discovery and validation sets. A) 5-oxoproline, B) L-octanoylcarnitine, C) docosahexaenoic acid, D) hypoxanthine.

In the discovery set, 70 plasma samples were analyzed and a total of 63 differential metabolites were identified. Table 2 shows their fold-change and area under the curve (AUC) values. Thirty-six metabolites showed significant fold-changes in the comparison between the cancer and healthy groups (*P* < 0.05); however, most of these metabolites were influenced by other physiological or metabolomical factors, which reduced their utility as biomarkers. After excluding all such metabolites, four (L-octanoylcarnitine, 5-oxoproline, hypoxanthine, and docosahexaenoic acid) were identified as candidate biomarkers of breast cancer. The AUCs of those four metabolites were significantly greater than 0.80 in the discovery set (*p* < 0.05) (Fig 2).

**Table 2:** Fold-changes of 63 metabolites and their AUC values.

Metabolite	Fold- change	AUC	Metabolite	Fold- change	AUC	Metabolite	Fold- change	AUC
**Amino acid**			**Nucleotides**			L-palmitoylcarnitine	1	0.416
Creatinine	0.66***	0.745	Hypoxanthine	7.62***	0.859	Dodecanoylcarnitine	0.40***	0.887
Sarcosine	1.32**	0.716	Uric acid	0.93*	0.680	Oleamide	0.96	0.568
5-oxoproline (pyroglutamic acid)	2.12***	0.968	Uridine	0.91	0.599	Palmitic amide	0.98	0.544
L-Threonine	1.08	0.527	**Organic compounds**			13Z-docosenamide	0.86	0.572
Betaine	0.92	0.571	Acetylcholine	0.76**	0.734	Suberic acid	1.02	0.62
Histidine	0.99	0.535	Phenylsulfate	2.12	0.510	Octanoic acid (caprylic acid)	1.62***	0.802
L-Lysine	0.94	0.543	Salicylaldehyde	1.13	0.595	Myristic acid	1.07	0.512
Homocysteinesulfinic acid	1.02	0.393	Trigonelline	3.14**	0.713	Palmitic acid	0.87*	0.654
L-phenylalanine	1.30***	0.838	Taurine	1.11	0.583	LysoPC(16:0)	1.25***	0.833
Indoxyl sulphate	2.74	0.642	3-Methoxy-4-hydroxyphenylethyleneglycol sulfate	1.33	0.597	LysoPC(16:1)	1.26*	0.683
L-Tryptophan	1.15	0.583	p-Cresol sulfate	0.47***	0.746	LysoPC(18:0)	1.34**	0.713
L-Isoleucine/Leucine	1.32**	0.728	Pyrocatechol sulfate	1.08	0.487	LysoPC(18:1)	1.18***	0.772
3-methyl-2-oxovaleric acid	0.91	0.579	Pyrogallol-2-O-sulphate	1.79	0.574	LysoPC(18:2)	1.42***	0.848
**Bile acid**			Bilirubin	0.22***	0.943	LysoPC(20:1)	1.17*	0.653
Glycoursodeoxycholic acid	2.62***	0.780	Oxoadipic acid	1.02	0.443	LysoPC(22:6)	1.47***	0.808
Glycochenodeoxycholic acid	4.46***	0.893	**Lipid**			LysoPC(O-16:0)	1.30***	0.761
Tauroursodeoxycholic acid	6.04***	0.892	Docosahexaenoic acid	0.53***	0.822	LysoPE(16:0)	1.15*	0.683
**Vitamins and Cofactors**			Cis-5-tetradecenoylcarnitine	0.43***	0.842	LysoPE(18:1)	1.97***	0.833
1-methylnicotimanide	0.56***	0.869	Linoleyl carnitine	1.35	0.593	LysoPE(18:2)	1.66***	0.795
**Carbohydrate**			L-octanoylcarnitine	0.41***	0.858	LysoPE(20:4)	1.34**	0.685
Glucose	1.29*	0.660	Vaccenyl carnitine	1.12	0.496	LysoPE(22:6)	1.1	0.577
**Energy**			L-acetylcarnitine	0.66***	0.784	Sphingosine-1-phosphate	1.65***	0.801
Isocitric acid/citric acid	0.62	0.614	L-carnitine	0.83*	0.697			

AUC; area under the curve

*p-value < 0.05

**p-value <0.01

***p-value <0.001.

### External validation of biomarker candidates

To validate the diagnostic performance of the four selected metabolites (L-octanoylcarnitine, 5-oxoproline, hypoxanthine, and DHA), the cut-off values obtained in the ROC analysis of the discovery set were applied to the participants of the validation set. L-octanoylcarnitine showed the highest positive predictive value (PPV), of 100.0%, while DHA showed a PPV of 91.3% (Table 3).

**Table 3:** External validation results of four candidate biomarkers.

	L-octanoylcarnitine	Docosahexaenoic acid	5-oxoproline	Hypoxanthine
Positive predictive value	100.0	91.3	86.4	76.0
Negative predictive value	47.1	60.9	54.2	47.6

To determine the reproducibility of the four metabolites as biomarkers in the validation set, we compared their mean ROC scores between the healthy and cancer groups: the scores for L-octanoylcarnitine and DHA were significantly lower in cancer patients compared to healthy controls, and vice versa for 5-oxoproline and hypoxanthine (Fig 2). The AUC values of the four metabolites were greater than 0.70 in the validation set (*P* < 0.05), indicating good reproducibility.

### Differences in metabolite profiles according to the pathological characteristic of tumors

**Fig 3:**
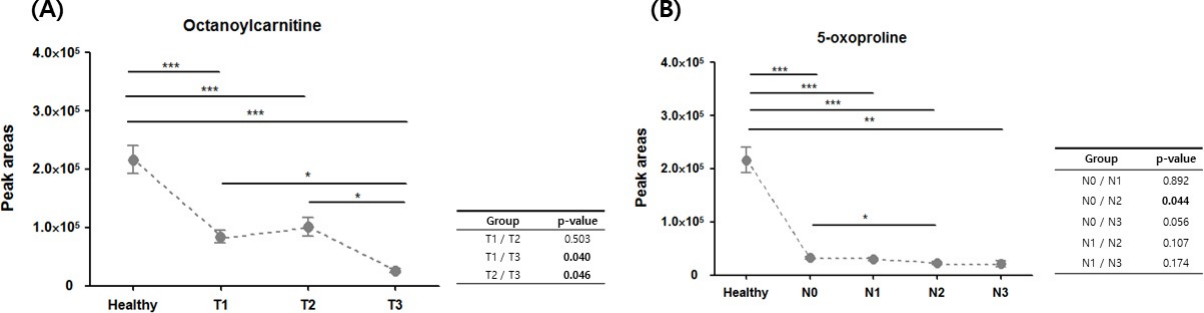
Differences in metabolite profiles according to tumor size and nodal stage in the discovery set. A) Octanoylcarnitine varied by tumor stage B) and 5-oxoproline varied by nodal stage.

**Fig 4:**
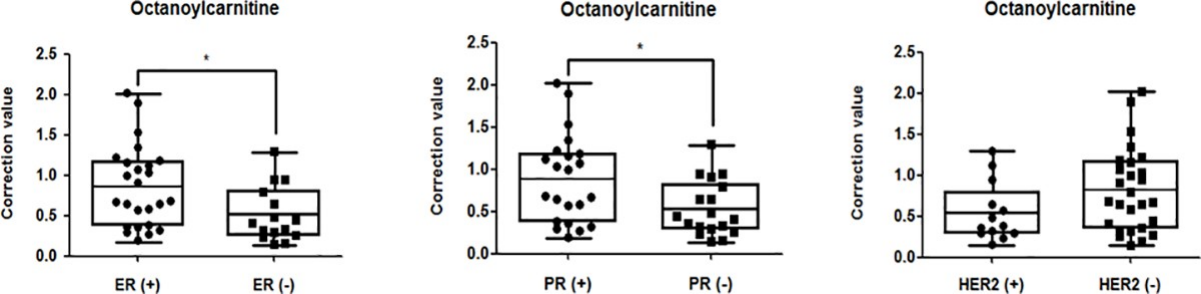
The octanoylcarnitine level was significantly higher in estrogen receptor (ER)- and progesterone receptor (PR)-expressing tumors, but no significant difference was observed according to HER-2 expression.

We then investigated whether the four metabolites differed according to the pathological characteristics of tumors in the discovery set. The octanoylcarnitine level was significantly lower in T3 tumors compared to T1 and T2 tumors (Fig 3). The 5-oxoproline levels in N2 and N3 tumors were lower compared to those in N0 tumors (Fig 3). Furthermore, the levels of octanoylcarnitine were significantly higher in estrogen receptor (ER)- and progesterone receptor (PR)-expressing tumors, but no significant difference was observed according to HER-2 expression (Fig 4). The other metabolites (hypoxanthine and DHA) showed no difference in expression according to tumor size, nodal status or hormone receptor expression.

## Discussion

Metabolomics is a useful approach for identifying biomarkers and metabolic alterations in cancer patients. Numerous studies have explored the possibility of metabolomic profiling for early detection of breast cancer [13–15].

Sitter et al. [13] distinguished between patients with good and poor prognoses based on analysis of a panel of metabolites in breast tumor tissues. Another study [16] analyzed blood samples collected from 56 surgically treated breast cancer patients using a combination of NMR and GC-MS; a panel of 11 markers predicted disease recurrence with a sensitivity of 86% and a specificity of 84%. Wei et al [17] reported that the serum metabolites threonine, isoleucine, glutamine and linolenic acid could be used as predictive markers of the response to neoadjuvant chemotherapy in breast cancer patients. Jobard et al. [18] analyzed blood samples collected from 197 early and 90 late-stage breast cancer patients using NMR, with the levels of histamine, alanine, and betaine being higher in the serum of the early stage group. Moreover, the early and late breast cancer patients could be distinguished with a sensitivity of 90% and specificity of 79%. The metabolites could be derived from tumors, host, or the microbiome. In breast cancer, metabolic changes in the host due to comorbidities such as obesity and diabetes might increase the risk of breast cancer and play a role in its progression. In addition, some metabolites derived from the gut microbiome could be involved in intracellular metabolism and molecular events in breast cancer, and/or affect the treatment thereof [19].

In the present study, we evaluated the possible utility of plasma metabolites as biomarkers for early diagnosis of breast cancer, via metabolomic analysis of plasma samples collected from 70 patients with breast cancer and 46 healthy subjects. We observed that the expression of several metabolites differed between breast cancer patients and healthy controls. Based on our analyses, four metabolites were selected as possible biomarkers of breast cancer. Among those four metabolites, the plasma levels of L-octanoylcarnitine and DHA were significantly lower in breast cancer patients. However, the levels of hypoxanthine and 5-oxoproline were significantly higher in the breast cancer patients.

We found that plasma levels of L-octanoylcarnitine were significantly lower in patients with breast cancer compared to the healthy controls, suggesting a high demand for carnitine for breast tumor metabolism. Carnitine is an important nutrient in food, particularly meat and dairy products. Carnitine has two principal functions in the organism: the first is to transport long-chain fatty acids into the mitochondria, and the second is to balance the acyl coenzyme A (CoA)-CoA ratio. This latter function is important because it allows removal of excessive (and potentially toxic) short- and medium-chain fatty acids from the mitochondria [20]. Against this background, our findings suggested that carnitine in cancer patients was involved in the transfer of long-chain fatty acids to the mitochondria. This explained the higher level of fatty acid beta-oxidation in breast cancer patients, consistent with recent studies reporting that lipolysis and lipid oxidation were upregulated in cancer cells [21, 22]. Fatty acids can fuel cancer cells, because mitochondrial fatty acid oxidation produces considerably more ATP than oxidation of other nutrients, such as glucose and amino acids [23]. These results also accorded with a recent study suggesting that the carnitine system is pivotal in the metabolic flexibility of cancer cells [24]. That report suggested that regulation of the carnitine system at both the enzyme and gene levels plays an important role in modulating the metabolic flux of tumors, which could be a promising target for new breast cancer therapies.

The omega-3 fatty acid DHA has anti-cancer effects. Several studies have indicated that DHA inhibits breast cancer cell growth and increases apoptosis [25–27]. The main mechanism underlying apoptosis induction via DHA is lipid peroxidation, which in turn increases reactive oxygen species (ROS) levels and activates caspase to induce caspases; this leads to apoptosis [28]. In the present study, DHA levels were significantly lower in patients with breast cancer compared to healthy controls, such that anti-cancer activity was lower in the patients.

Xanthine oxidase is the final enzyme involved in the degradation of purines; it converts hypoxanthine to xanthine and, subsequently, to uric acid, with ROS being generated as a byproduct [29]. Purine nucleotides are made available for cells via two routes, i.e., de novo synthesis or reuse of catabolized purine bases (mainly, hypoxanthine). The purine salvage pathway is more efficient in terms of ATP equivalents than de novo purine synthesis [30] and drives the growth of cancer cells by enabling more efficient production of ATP versus surrounding normal cells. In our study, high plasma levels of hypoxanthine in the breast cancer patients suggested that the purine biosynthesis pathway could have been overridden by the salvage pathway in breast cancer cells.

Pyroglutamic acid, also known as 5-oxoproline, is the cyclic lactam of glutamic acid. The role of pyroglutamic acid in living cells has not been well studied. In several genetic disorders, and in an acetaminophen-induced metabolic disorder, large amounts of pyroglutamic acid are secreted in the urine (i.e., 5-oxoprolinuria). Free pyroglutamic acid may play a role as an analogue or reservoir of glutamate [31]. Exogenous glutamine is an important source of energy and a molecular building block for many tumors [32]. The relationship between breast cancer and pyroglutamic acid has not yet been clarified, but the potential role of glutamine should be further investigated.

To validate the predictive value of the four biomarkers identified in this study, the cut-off values obtained from the ROC analysis of the discovery set were applied to the validation set. The four selected metabolites, L-octanoylcarnitine, 5-oxoproline, hypoxanthine, and DHA, showed potential as biomarkers for breast cancer, with PPVs greater than 75%. Taken together, these results indicated that metabolic profiling may be a promising approach for the identification of diagnostic biomarkers of breast cancer.

We also analyzed the relationship between these metabolites and hormone receptor status, and found significant differences in the expression of octanoylcarnitine according to ER and PR expression levels (Fig 4). One study reported that in breast cancer without ER expression, beta-alanine, 2-hydroyglutarate, glutamate, and xanthine levels were increased, and that of glutamine was decreased [33]. However, the relationship between octanoylcarnitine levels and hormone receptor status has not yet been reported, so further research is needed. The prognosis for women with breast cancer depends not only on early diagnosis, but also on the tumor size and lymph node metastasis status at the time of presentation [34]. Although several studies have examined metabolic alterations according to tumor stage, there has been minimal research on the relationship between tumor size and nodal metastasis [35, 36]. Importantly, there has been no report on metabolites such as 5-oxoprolinuria and L-octanoylcarnitine, which showed differences by tumor size and lymph node metastasis in this study. Based on our results, it may be possible to predict the prognosis and likelihood of recurrence of breast cancer via metabolomic analysis. The major limitation of this study was the relatively small sample size. Therefore, the diagnostic performance of metabolites for breast cancer still needs to be confirmed in further studies including larger sample sizes.

## Conclusion

We showed that the concentrations of plasma metabolites, such as L-octanoylcarnitine, 5-oxoproline, hypoxanthine, and DHA, differed between breast cancer patients and healthy controls; these metabolites showed high PPVs for breast cancer. Thus, the results suggested that plasma metabolites are promising candidates for early diagnosis of breast cancer. Furthermore, the collection of blood samples alone might allow for early screening of breast cancer patients, which points to the possibility of mass screening.

## Supporting information

S1 File Data. (ZIP)
